# Novel Reversible Inhibitors of Xanthine Oxidase Targeting the Active Site of the Enzyme

**DOI:** 10.3390/antiox12040825

**Published:** 2023-03-28

**Authors:** Rosario Rullo, Carmen Cerchia, Rosarita Nasso, Virgilio Romanelli, Emmanuele De Vendittis, Mariorosario Masullo, Antonio Lavecchia

**Affiliations:** 1 Institute for the Animal Production Systems in the Mediterranean Environment, CNR, 80055 Portici, Italy; 2 Department of Molecular Medicine and Medical Biotechnology, University of Naples Federico II, 80131 Naples, Italy; 3 Department of Pharmacy, “Drug Discovery” Laboratory, University of Naples Federico II, 80131 Naples, Italy; 4 Department of Human Movement Sciences and Wellness, University of Naples “Parthenope”, 80133 Naples, Italy

**Keywords:** xanthine oxidase, xanthine oxidase inhibitors, drug discovery, kinetic studies, redox enzymes

## Abstract

Xanthine oxidase (XO) is a flavoprotein catalysing the oxidation of hypoxanthine to xanthine and then to uric acid, while simultaneously producing reactive oxygen species. Altered functions of XO may lead to severe pathological diseases, including gout-causing hyperuricemia and oxidative damage of tissues. These findings prompted research studies aimed at targeting the activity of this crucial enzyme. During the course of a virtual screening study aimed at the discovery of novel inhibitors targeting another oxidoreductase, superoxide dismutase, we identified four compounds with non-purine-like structures, namely **ALS-1**, **-8**, **-15** and **-28**, that were capable of causing direct inhibition of XO. The kinetic studies of their inhibition mechanism allowed a definition of these compounds as competitive inhibitors of XO. The most potent molecule was **ALS-28** (*K*i 2.7 ± 1.5 µM), followed by **ALS-8** (*K*i 4.5 ± 1.5 µM) and by the less potent **ALS-15** (*K*i 23 ± 9 µM) and **ALS-1** (*K*i 41 ± 14 µM). Docking studies shed light on the molecular basis of the inhibitory activity of **ALS-28**, which hinders the enzyme cavity channel for substrate entry consistently with the competitive mechanism observed in kinetic studies. Moreover, the structural features emerging from the docked poses of **ALS-8**, **-15** and **-1** may explain the lower inhibition power with respect to **ALS-28**. All these structurally unrelated compounds represent valuable candidates for further elaboration into promising lead compounds.

## 1. Introduction

Xanthine oxidase (XO) and xanthine dehydrogenase (XDH) are different cytosolic forms of a single gene transcript named xanthine oxidoreductase (XOR) [1]. In humans, both forms of this enzyme display a crucial role in purine catabolism, being involved in the homeostasis of several redox species, including xenobiotics [2,3,4]. XDH and XO are composed of two identical subunits, each of them consisting of three domains: an N-terminal domain containing two iron–sulphur (Fe-S) clusters, a central domain with the flavine adenine dinucleotide (FAD) and a C-terminal domain containing a molybdopterin cofactor [5]. XDH and XO catalyse the oxidation of hypoxanthine to xanthine and then to uric acid by transferring electrons to the molybdenum centre and subsequently to FAD, thanks to the mediation of the two Fe-S centres located on the enzyme. The electrons are finally accepted by NAD^+^ or O_2_ in the XDH- or XO-catalysed reaction, respectively [5,6,7,8,9]. In physiological conditions, the predominant form of the enzyme is XDH, whereas XO becomes more abundant in oxidant environments. The conversion of XDH to XO may occur through the reversible oxidation of some cysteine residues of XDH to form disulphide bridges, or via an irreversible limited proteolysis of XDH [10,11,12,13,14,15]. Mainly in the XO-catalysed reaction are a significant amount of reactive oxygen species (ROS) formed via the univalent or divalent reduction of O_2_ to O_2_^•–^ or H_2_O_2_, respectively [7,16]; these latter being produced even during the XDH-catalysed reaction [6].

The overexpression and/or hyperactivity of XO produces an increased amount of uric acid, a recognised risk factor for gout [17,18,19]. Gout is a very painful form of arthritis associated with inflammation. It is primarily caused by the deposition of monosodium urate crystals in joints due to increased serum uric acid levels, triggering recurrent episodes of pronounced acute inflammation, known as gout flares [20]. There are generic and metabolic risk factors for gout, and multiple comorbidities, such as metabolic syndrome and cardiovascular and renal diseases.

Furthermore, the increased levels of ROS triggered by the upregulation of XO cause oxidative damage to living tissues, thus leading to other diseases linked to oxidative stress, such as inflammation, cardiovascular diseases, heart failure, hypertension, atherosclerosis, renal hypoxia, diabetes, metabolic syndrome and carcinogenesis [9,19,21,22,23,24,25,26,27,28]. For this reason, for several years, scientific attention has been focused on the identification of inhibitors of XO, which could be used to reduce the increased levels of uric acid and ROS in humans, thus contrasting the above-mentioned diseases [3,9,18,29,30,31]. 

For instance, pharmacological XO inhibition has been reported to be beneficial for the prevention of cardiovascular events: a recent meta-analysis compared the incidence of major adverse cardiovascular events, mortality and total and specific cardiovascular events in randomised controlled trials evaluating XO inhibitors against placebo or no treatment [32]. This study concluded that XO inhibition may reduce the incidence of adverse CV outcomes, with the beneficial outcomes linked to both the antioxidant effects (resulting from the inhibition of ROS production) and the reduction of uric acid levels. In fact, uric acid fosters an inflammatory state by promoting the growth of vascular smooth cells, activating the renin–angiotensin system and decreasing nitric oxide. A systemic inflammatory state and oxidative stress are also the hallmarks of chronic inflammatory diseases, such as rheumatoid arthritis, lupus and psoriasis. Chronic inflammatory diseases, in turn, are associated with an augmented risk of developing cardiovascular diseases, likely correlated with the underlying increase in several pro-inflammatory cytokines, such as TNF, IL-1 and IL-17, IL-6 [33]. As a result, mediators of articular and cutaneous inflammation may also be involved in metabolic and atherosclerosis disease, which may lead to comorbidities. 

By contrast, uric acid has been found to exert a protective effect against peroxynitrite-related nitration in the heart [34]. Thus, low uric acid levels have potential protective effects by reducing tissue injury mediated by peroxynitrite.

**Figure 1:**
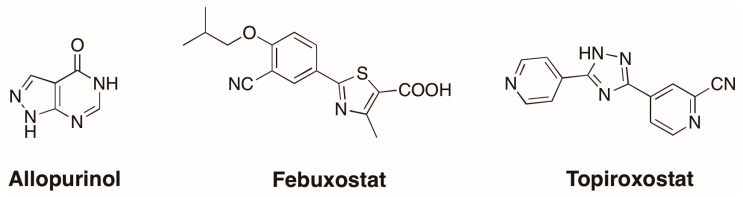
Chemical structures of approved XO inhibitors allopurinol, febuxostat (TEI-6720), topiroxostat (FYX-051).

The first identified inhibitor of XO was allopurinol (Figure 1), a compound with a purine-like structure. This molecule received Food and Drug Administration (FDA) approval and has been widely used for the treatment of gout, although it has some serious side-effects [35,36,37,38]. Other compounds with a non-purine structure, such as febuxostat [39] and topiroxostat [40], have been proposed and approved (Figure 1); however, also in this case, some side-effects have been reported. An investigation on the inhibition mechanism possessed by TEI-6720 (febuxostat) showed that this compound has a mixed-type inhibition, probably because of its tight binding to the molibdopterin cofactor of XO, which hampered the entry of the substrate into the active site of the enzyme [41]. Similarly, FYX-051 (topiroxostat) displayed a hybrid-type inhibition mechanism, forming a covalent linkage with the active site of the enzyme [42]. More recently, another compound extracted from plants, fraxamoside, has been identified as an unusual XO inhibitor [43,44]. 

In a previous virtual screening study, we identified small molecules targeting another antioxidant enzyme, namely superoxide dismutase from *Streptococcus mutans* (*Sm*SOD) [45,46], which catalyses the ROS produced by the activity of XO. During this study, we identified four structurally unrelated molecules, with different non-purine scaffolds, capable of causing direct inhibition of XO. The kinetic studies on the XO-catalysed reaction demonstrated that these compounds act as competitive inhibitors of XO, with the most potent compound, **ALS-28**, displaying a *K*_i_ value of 2.7 µM. Docking studies showed that these compounds are well positioned within the active site of XO and obstruct the cavity channel for substrate entry, a finding that is in agreement with the competitive mechanism observed in kinetic studies. Therefore, **ALS-28** and the other identified XO inhibitors may represent valuable candidates for further elaboration into promising lead compounds [47,48,49].

## 2. Materials and Methods

### 2.1. Materials and Reagents

Compounds **ALS-1**, **-8**, **-15** and **-28**, included in a list of possible inhibitors of *Sm*SOD [46], were purchased from Otava (http://www.otavachemicals.com/, accessed on 28 September 2015). These compounds were at least 90% pure and stock solutions were prepared in dimethylsulfoxide (DMSO) at a 20 mM concentration. Xanthine, xanthine oxidase from bovine milk (0.5 U/mg) and all other reagents and solvents of high analytical grade were purchased from Sigma-Aldrich (St. Louis, MO, USA).

### 2.2. Computational Methods

#### 2.2.1. Protein and Ligand Preparation

The crystal structure of bovine xanthine oxidase in complex with febuxostat (PDB 1N5X) [41] was retrieved from the Protein Data Bank (PDB). The amino acid sequence has 90% sequence identity to the human form of the enzyme [50].

The structure was preprocessed and optimised with the Protein Preparation Wizard in Maestro (Protein Preparation Wizard; Epik, Schrödinger, LLC, New York, NY, USA, 2021; Impact, Schrödinger, LLC, New York, NY, USA; Prime, Schrödinger, LLC, New York, NY, USA, 2021). The hydrogen atoms were added after determining the appropriate bond orders, charges and atom types. Extensive sampling of the rotamers, tautomers and protonation states of titratable amino acids at neutral pH was performed in order to optimise the H-bond network.

Finally, the protein structure was subjected to a restricted minimisation using the Impref module and the OPLS4 force field, with a 0.3 RMSD limit imposed from the original coordinates as a constraint.

Compounds under investigation, namely **ALS-1**, **-8**, **-15** and **-28**, were drawn by means of a Maestro 2D-sketcher and prepared with LigPrep (LigPrep, Schrödinger, LLC, New York, NY, USA, 2021) to generate suitable 3D conformations and tautomerisation states at pH 7.0 ± 2.0. The compounds were then energetically minimised using the OPLS4 force field.

#### 2.2.2. Docking Simulations

Docking of compounds under study was performed with the Glide algorithm (Glide, Schrödinger, LLC, New York, NY, USA, 2021) in a Standard Precision (SP) mode [51,52]. For the docking grid generation, an inner box of 20 × 20 × 20 Å, surrounding the febuxostat binding cavity site was considered. Van der Waals radii of receptor atoms were scaled at a factor of 0.8. Flexible ligand sampling was permitted, and no constraints were applied. Default docking parameters were used when not specified. The GlideScore function was used to score and rank the predicted binding poses. For each ligand, ten poses were generated; the final docked poses were selected on the basis of the scoring, the similarity to the co-crystallised ligand binding mode and the consistency of protein–ligand interactions with the experimental data. In order to ensure the reliability of the docking simulations, pose generation quality was first investigated by re-docking of the co-crystalised ligand febuxostat. The docking protocol well reproduced the experimental geometries, with root-mean-square deviation (RMSD) values of less than 2 Å for all the ten generated poses. Figures were rendered with PyMOL (The PyMOL Molecular Graphics System, Version 2.0 Schrödinger, LLC).

### 2.3. Biochemical Methods

The activity of XO was measured spectrophotometrically at 25 °C by monitoring the increase of absorbance at 295 nm due to the formation of uric acid, using a Cary 100 UV-Vis Spectrophotometer (Agilent Technologies, Milan, Italy), essentially, as previously reported [44,53]. In steady-state determination of the XO activity, each assay was carried out in a 500 µL final volume reaction mixture, containing 100 mM phosphate buffer, pH 7.8 plus 0.1 mM EDTA (buffer A), 75 µM xanthine and different concentrations of the XO inhibitors. The DMSO concentration carried over by the inhibitor was 0.5% (*v*/*v*); an identical concentration of DMSO was used in the absence of inhibitor. The reaction started with the addition of XO and was followed kinetically up to 30 s by continuously monitoring the absorbance. The initial rate of uric acid formation was derived from the linear part of the kinetics and expressed as ΔE/min. The effect of each inhibitor was evaluated through the ratio of residual XO activity measured in the presence of each inhibitor over that measured in the absence of inhibitor. The concentration of inhibitor leading to 50% reduction of XO activity (IC_50_) was calculated from a logarithmic transformation of the reported residual XO activity vs. the inhibitor concentration in a semilogarithmic plot.

The reversibility of the inhibition mechanism was evaluated through a dilution method [54,55,56] of a solution containing XO and inhibitor. To this aim, a sample of XO was incubated at 25 °C in the absence or presence of a concentrated solution of inhibitor. Aliquots of these mixtures were withdrawn at different times and 4-fold diluted through the addition of a solution containing the substrate xanthine. The samples were then immediately assayed for XO activity as previously indicated for the steady-state determinations.

The inhibition power of the various compounds and their mechanism of inhibition were assessed through kinetic measurements of the XO activity. To this aim, each assay was carried out in a 500 µL final volume reaction mixture, containing 4–30 µM xanthine dissolved in buffer A in the absence or presence of a fixed concentration of the various inhibitors. The reaction started with the addition of XO and was followed as indicated in steady-state assays. The kinetic parameters of the reaction, *K*_M_ for the substrate and *V*_max_ of the reaction, were derived either from the direct non-linear interpolation in the Michaelis–Menten hyperbolic equation of the initial rate of uric acid formation vs. the xanthine concentration or from a double reciprocal transformation of the kinetic data in Lineweaver–Burk plots. According to a reversible competitive inhibition mechanism, the *V*_max_ of the reaction remained essentially unvaried in the presence of the tested inhibitors, whereas the *K*_M_ for xanthine significantly increased. Therefore, the inhibition constant (*K*_i_) was obtained from the increase of *K*_M_ for xanthine in the presence of the inhibitor, according to the *K*_M_′ = *K*_M_•{1 + ([I]/*K*_i_)} equation, where *K*_M_′ represents the *K*_M_ for xanthine measured in the presence of the concentration [I] of the inhibitor.

### 2.4. Statistical Analysis

Data were analysed using the KaleidaGraph program (Synergy, 5.0 version, Adalta, Italy) and reported as the mean ± standard error (SE). The statistical significance of non-linear and linear fittings of the data was evaluated with the correlation coefficient *R*, which was always >0.960.

## 3. Results and Discussion

### 3.1. Novel Inhibitors of Xanthine Oxidase with Non-Purine-Like Structures

Xanthine oxidase (XO) is an enzyme frequently used in the measurement of the activity of another enzyme, superoxide dismutase (SOD), through the inhibition of cytochrome *c* reduction caused by superoxide anions generated with the xanthine/xanthine oxidase method [57,58]. This indirect assay was chosen to evaluate the possible inhibition of SOD from *Streptococcus mutans* (*Sm*SOD) by thirty-six small molecules (**ALS-1**→**36**, Table S1, Supplementary Materials) selected through a virtual screening method [46]. Briefly, we established two main approaches, structure-based (SBVS) and ligand-based (LBVS) virtual screening. The first approach aimed at disrupting the enzyme dimer interface, whose integrity is critical for *Sm*SOD activity. Overall, the SBVS approach provided ten compounds (Table S1). On the other hand, the LBVS aimed at the identification of ligands furnished with functional groups known to potentially act as metal chelators, such as carboxylic acid, tetrazole and pyridine moieties, since the metal centre has a key role in *Sm*SOD catalysis and thus any alteration in the active site by metal chelation or modifications in the coordination geometry could affect the enzyme’s antioxidant action and, therefore, influence both the growth and survival of the pathogen. The LBVS approach provided 26 compounds in total (Table S1).

**Figure 2:**
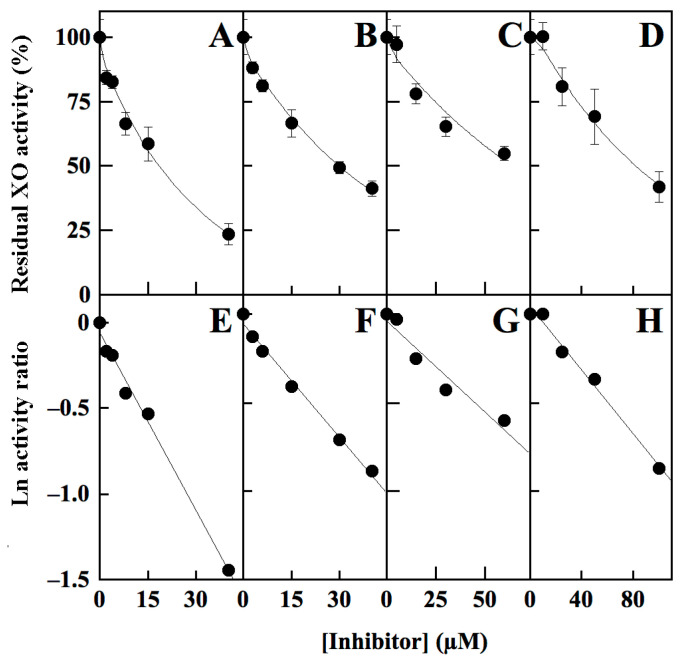
Dose-dependent inhibition profile of xanthine oxidase activity by **ALS-28**, **-8**, **-15** or **-1**. The activity of XO (12.5 mU) was measured in buffer A in the presence of 75 µM xanthine through the initial rate of uric acid formation in the presence of the indicated concentration of **ALS-28** (panels (**A**) and (**E**)), **ALS-8** (panels (**B**) and (**F**)), **ALS-15** (panels (**C**) and (**G**)) or **ALS-1** (panels (**D**) and (**H**)). The activity was expressed either as a percentage of that measured in the absence of inhibitor (panels (**A**)–(**D**)) or as a natural logarithm of the ratio of residual activity (panels (**E**)–(**H**)). Values were reported as the mean ± SE (panels (**A**)–(**D**)); in the logarithmic representation of the data (panels (**E**)–(**H**)), the correlation coefficients *R* ranged in the 0.966–0.995 interval. Other details are described in Materials and Methods.

However, four out of these 26 compounds (namely, **ALS-1**, **-8**, **-15** and **-28**) were excluded from the analysis because they affected the activity of XO in the absence of SOD [46]. This observation prompted an evaluation of whether these compounds could act as putative XO inhibitors. To this aim, a direct assay, based on the conversion of xanthine to uric acid, catalysed by XO, was used for evaluating their effects on the enzyme from bovine milk (Figure 2). **ALS-28** (Figure 2A), **ALS-8** (Figure 2B), **ALS-15** (Figure 2C) and **ALS-1** (Figure 2D), although with a different inhibition strength, caused a dose-dependent inhibition of XO. 

A logarithmic transformation of the data of residual XO activity allowed the calculation of the IC_50_ values for **ALS-28** (Figure 2E), **ALS-8** (Figure 2F), **ALS-15** (Figure 2G) and **ALS-1** (Figure 2H). These parameters of the inhibition power, reported for each compound in Table 1, indicate that **ALS-28** displayed the strongest effect (18 µM), followed by **ALS-8** (30 µM), **ALS-15** (64 µM) and **ALS-1** (82 µM), in that order.

### 3.2. Mechanism of XO Inhibition

**Figure 3:**
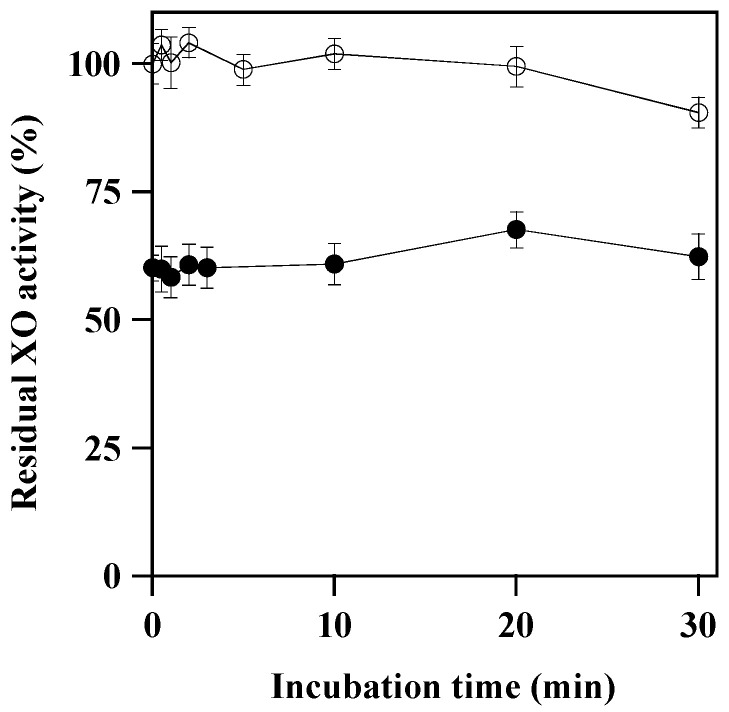
Reversibility of the inhibition by **ALS-28** in xanthine oxidase samples incubated at 25 °C. A sample of XO (0.136 U/L in buffer A) was incubated at 25 °C in the absence (open circles) or presence of 60 µM **ALS-28** (filled circles). At the indicated times, 125 µL aliquots were withdrawn and then immediately assayed for XO activity after the addition of 375 µL of 100 µM xanthine in buffer A. The residual XO activity was expressed as a percentage of that measured in the absence of inhibitor at time zero. Values were reported as the mean ± SE. Other details are described in Materials and Methods.

The reversibility of the inhibition mechanism was investigated through a dilution method. To this aim, samples of XO were incubated at 25 °C at different times in the absence or presence of 60 µM **ALS-28**, and then immediately assayed for activity after a 4-fold dilution of the samples, thus reducing the inhibitor concentration to 15 µM. As shown in Figure 3, no time-dependent reduction of XO activity was observed up to 30 min, for either untreated or treated enzyme. Furthermore, at the beginning of the preincubation between XO and inhibitor, the level of residual activity measured in the presence of 15 µM **ALS-28** was essentially coincident with that reported in Figure 2, where no preincubation was applied, and remained almost constant up to 30-min incubation. These findings indicate that **ALS-28** acts as a reversible inhibitor, because no differences in the inhibition power were observed within the 30-min period time. A similar behaviour was obtained with the less potent inhibitor **ALS-15** (data not shown).

**Figure 4:**
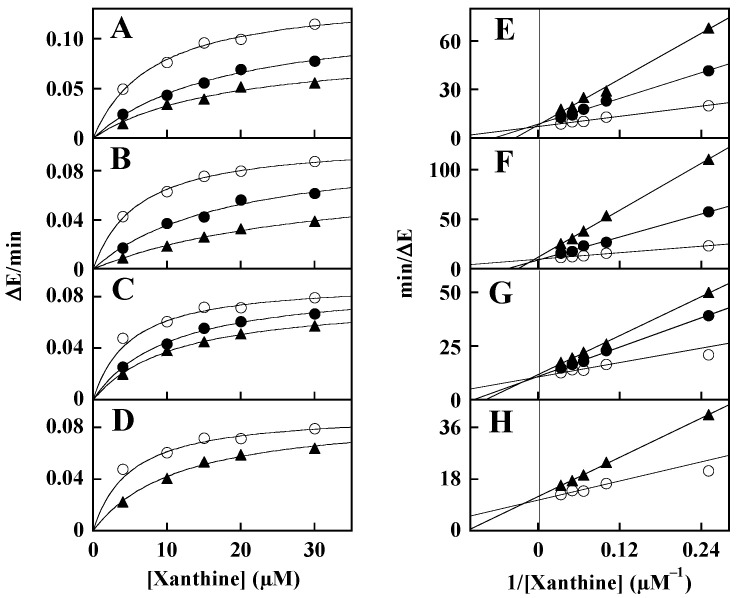
Kinetic analysis of the xanthine oxidase inhibition by **ALS-28**, **-8**, **-15** or **-1**. The XO activity (12.5 mU) was measured in the presence of 4–30 µM xanthine through the initial rate of uric acid formation in the absence (open circles) or presence (filled symbols) of fixed concentrations of the various inhibitors. Panels (**A**) and (**E**), effect of 3 µM (filled circles) or 10 µM (triangles) **ALS-28**. Panels (**B**) and (**F**), effect of 7.5 µM (filled circles) or 25 µM (triangles) **ALS-8**. Panels (**C**) and (**G**), effect of 12 µM (filled circles) or 30 µM (triangles) **ALS-15**. Panels (**D**) and (**H**), effect of 30 µM (triangles) **ALS-1**. Data were reported using the hyperbolic Michaelis–Menten equation (panels (**A**)–(**D**)) or the linear Lineweaver–Burk representation (panels (**E**)–(**H**)). The correlation coefficients *R* ranged in the interval 0.966–0.998 (non-linear fit the Michaelis–Menten equation) or 0.966–0.999 (linear fit the Lineweaver–Burk plot); other details are described in Materials and Methods.

The inhibition mechanism of **ALS-28**, **-8**, **-15** and **-1** was better evaluated through kinetic measurements of XO activity. In particular, the time-dependent formation of uric acid was measured at different xanthine concentration in the absence or presence of various inhibitor concentrations. The resulting data of initial velocity were analysed either in the typical Michaelis–Menten representation (Figure 4A–D) or in Lineweaver–Burk plots (Figure 4E–H), thus allowing an inspection of the inhibition mechanism displayed by **ALS-28** (Figure 4A,E), **ALS-8** (Figure 4B,F), **ALS-15** (Figure 4C,G) and **ALS-1** (Figure 4D,H). The affinity of XO for xanthine measured in the absence of inhibitor (*K*_M_ = 5.9 ± 1.2 μM) significantly increased in the presence of all inhibitors, whereas the maximum velocity of the reaction (*V*_max_ = 0.107 ± 0.021 ΔE/min in the absence of inhibitor) remained essentially unvaried in the presence of the different concentrations of each inhibitor (Table 2). This behaviour corresponds to a typical competitive inhibition of XO and therefore **ALS-28**, **-8**, **-15** and **-1** may be classified as competitive inhibitors of XO. The corresponding *K*_i_ values calculated for each compound reported in Table 2 indicate that **ALS-28** (*K*_i_ = 2.7 ± 1.5 μM) is endowed with the strongest inhibition power, followed by **ALS-8** (*K*_i_ = 4.5 ± 1.5 μM), whereas **ALS-15** (*K*_i_ = 23 ± 9 μM) and **ALS-1** (*K*_i_ = 41 ± 14 μM) both have a much lower inhibition power. The lower *K*_i_ compared to IC_50_ values reported in Table 1 may be explained with the competitive inhibition mechanism displayed by the four inhibitors, leading to an apparently lower inhibition power shown by the inhibitors when assayed at saturating substrate concentration (see Figure 2). Interestingly, the four compounds possess different structures, as shown in Table 1. From the structural point of view, these compounds qualify as small molecular fragments (MW < 300  Da), representing a model structure for a lead optimisation program aimed at improving the potency and specificity of their inhibition.

In particular, **ALS-1-**, **-8** and **-28** share a carboxylic acid group linked to a heterocycle core, (indole, pyrazole and pyrimidine, respectively); **ALS-15**, instead, features a tetrazole ring, which can be considered a carboxylic acid moiety bio-isostere.

### 3.3. Docking Studies

**Figure 5:**
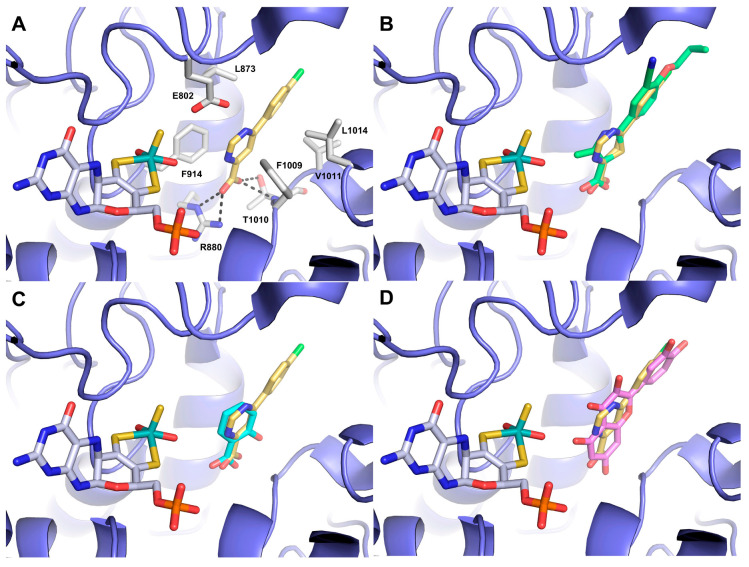
Predicted binding mode of compound **ALS-28** ((**A**), yellow sticks) into XO (slate ribbons, PDB 1N5X). Only amino acids discussed in the main text are displayed (white sticks) and labelled. H-bonds discussed in the text are depicted as dashed black lines. Overlay of **ALS-28** docked pose on febuxostat ((**B**), green sticks, PDB 1N5X), salicylic acid ((**C**), cyan sticks, PDB 1FIQ) and quercetin ((**D**), violet sticks, PDB 3NVY) co-crystallised into XO.

In order to shed light on the potential binding mode of compounds **ALS-28**, **-8**, **-15** and **-1** and to aid the interpretation of SAR data, we undertook docking studies using the X-ray structure of XO in complex with the FDA-approved inhibitor TEI-6720 (febuxostat) [41]. The top-ranked docking pose showed that **ALS-28**, the most potent compound, was well positioned within the enzyme active site, adjacent to the molybdopterin cofactor, where the substrate binds and is oxidised (Figure 5A). The carboxylate moiety engaged a strong salt bridge with R880 and two H-bonds with the side chain and the NH main chain of T1010. These residues are known to contribute to the enzyme’s catalytic mechanism [31]. In addition, the pyrimidine ring is sandwiched between two phenylalanine residues, F914 and F1009, which form a parallel and T-shaped p–p stacking interaction, respectively. The *p*-Cl-phenyl ring contributes further hydrophobic interactions with L1014, V1011 and L873. The overall binding mode revealed that the bound inhibitor hinders the cavity channel, thus blocking the binding of the substrate and its movement towards the metal complex, which is consistent with the competitive mechanism observed in kinetic studies. The docked pose of **ALS-28** displayed a good overlap with the crystallographic pose of febuxostat (Figure 5B), with a particular regard to their carboxylic acid moieties and the heterocyclic scaffolds. A nearly perfect fit could also be observed with salicylic acid (Figure 5C), which acts as a competitive inhibitor. Another inhibitor classified as competitive is quercetin: its crystallographic pose showed a similar positioning to **ALS-28**, even though quercetin approaches more closely to the molybdopterin cofactor (Figure 5D). 

**Figure 6:**
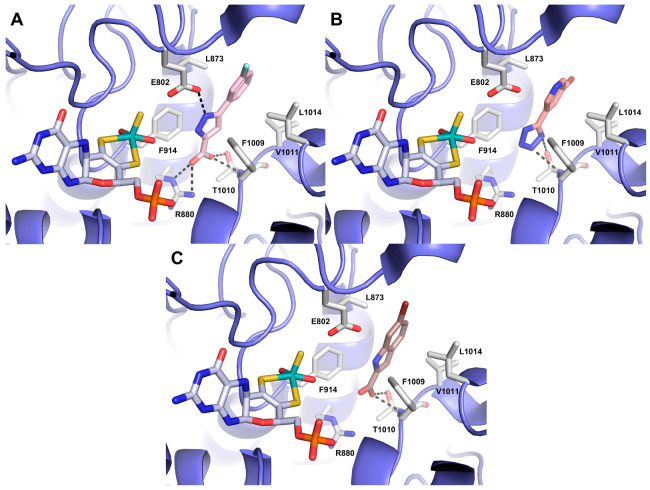
Predicted binding modes of compounds **ALS-8** ((**A**), pink sticks), **ALS-15** ((**B**), salmon sticks) and **ALS-1** ((**C**), dirty violet sticks) into XO (slate ribbons, PDB 1N5X). Only amino acids discussed in the main text are displayed (white sticks) and labelled. H-bonds discussed in the text are depicted as dashed black lines.

Compound **ALS-8** showed an identical interaction pattern to **ALS-28** (Figure 6A), mainly driven by the presence of the acidic group, a heterocyclic core (which, in the case of **ALS-8**, is a pyrazole ring) and a halogen-substituted phenyl ring. **ALS-15** was ranked as a much less powerful inhibitor, which could be due to the absence of the carboxylic acid function, which is able to strongly anchor the inhibitor within the binding cavity. This compound interacted mainly with T1010 (Figure 6B), while also maintaining stacking interactions with F914 and F1009. On the other hand, **ALS-1**, still bearing the acidic group at position 2 of an indole scaffold, behaved as the weakest inhibitor of the series. **ALS-1** interacted more strongly with T1010 (Figure 6C), whereas the salt bridge with R880 was weakened by a longer distance and the stacking interactions with F914 and F1009 were lost, thus explaining the drop of potency compared to **ALS-28** and -**8**. Hence, a heterocycle core (such as pyrimidine or pyrazole, resembling the thiazole moiety of febuxostat) linked to a phenyl ring seems to be preferred over an enlarged aromatic moiety such as the indole. The carboxylic acid function is, by consensus, a crucial pharmacophoric feature because of its formation of strong H-bonds with the key residues R880 and T1010, located within the active pocket, as also reported in literature [9]. The reversible and competitive inhibition mechanism confirmed by the docking studies is an important issue of this work, and could be useful for a deeper focus on this category of XO inhibitors.

## 4. Conclusions

In response to the established involvement of XO in a number of diseases linked to an elevated production of uric acid and ROS, several studies have been carried out using this essential antioxidant flavoenzyme as a target of small molecule inhibitors. 

Among the FDA-approved compounds targeting XO, allopurinol, febuxostat and topiroxostat are surely beneficial for treating specific diseases; however, they have been reported to cause, in some cases, severe undesired side-effects. For instance, allopurinol and other purine derivatives have been associated with gastrointestinal distress, renal toxicity, rash and eosinophilia, hampering their broader therapeutic use [3,18]. Febuxostat recently received a black box warning from the FDA due to the increased risk of cardiovascular mortality, thus limiting the use of febuxostat to patients for whom allopurinol is not efficacious or patients who experience severe adverse effects with allopurinol was recommended. 

Therefore, the identification of novel compounds acting as powerful inhibitors of XO without causing adverse reactions represents an important scientific aim. More recently, one of the main objectives in the design of XO inhibitors has been the development of novel non-purine-like inhibitors [9,29,30,31,50]. In this regard, the compounds **ALS-28**, **-8**, **-15** and **-1** could be valuable candidates as promising lead compounds, because all these structurally unrelated molecules have a non-purine structure. Among them, the most powerful compounds, **ALS-28** and **ALS-8**, are endowed with a convenient inhibition strength, *K*_i_ 2.7 µM and 4.5 µM, respectively, and are therefore appropriate for further improvements in potency through future optimisation programs. The other two compounds, **ALS-15** and **ALS-1,** displayed lower potency; docking studies highlighted some structural features probably responsible for their reduced efficiency as XO inhibitors. Lastly, these compounds showed a reversible and competitive mechanism of inhibition in kinetic studies, which is an additional intriguing aspect, because this category is generally regarded as a safer therapeutic option. Given this concern, the possible toxicity of these molecules in a cellular context should be investigated as the first step of a future research plan aimed at considering the downregulation of eventually altered XO functions. As an example, the research should exclude any toxic effects by these compounds on the cell viability of ubiquitous non-malignant cell lines.

In conclusion, the herein presented compounds may represent the starting point to develop novel families of powerful inhibitors of XO, an oxidoreductase involved in the homeostasis of redox species, that, in some cases, may lead to severe pathological diseases.

## Figures and Tables

**Table 1:** Values of IC_50_ obtained from inhibition profiles of structurally unrelated inhibitors of xanthine oxidase.

Inhibitor	Structure	Steady-State Determination ^(a)^	
		Concentration Interval of the Inhibitor	IC_50_ µM
ALS-28	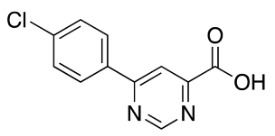	2–40 µM	18
ALS-8	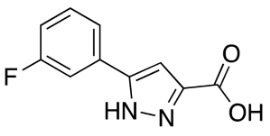	3–40 µM	30
ALS-15	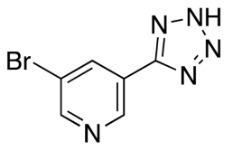	5–60 µM	64
ALS-1	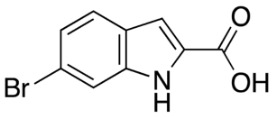	10–100 µM	82

^(a)^ Measurements performed in the presence of 75 µM xanthine.

**Table 2:** Kinetic parameters and inhibition constants of the novel xanthine oxidase inhibitors.

Inhibitor	Kinetic Determination^*a*^			
	Concentration of the Inhibitor (µM)	*K*_M_ (µM)	*V*_max_ (ΔE/min)	*K*_i_ (µM)
None		5.9 ± 1.2 (*n =* 8)	0.107 ± 0.021 (*n = 8*)	
**ALS-28**	3	16.8 ± 1.5 (*n =* 2)	0.113 ± 0.007 (*n =* 4)	2.7 ± 1.5 (*n =* 4)
	10	23.2 ± 5.1 (*n =* 2)		
**ALS-8**	7.5	19.8 ± 1.4 (*n =* 2)	0.093 ± 0.007 (*n =* 4)	4.5 ± 1.5 (*n =* 4)
	25	31.8 ± 2.0 (*n =* 2)		
**ALS-15**	12	10.3 ± 2.0 (*n =* 2)	0.086 ± 0.003 (*n =* 4)	23 ± 9 (*n =* 4)
	30	11.9 ± 0.8 (*n =* 2)		
**ALS-1**	30	10.5 ± 1.1 (*n =* 2)	0.087 ± 0.004 (*n =* 2)	41 ± 14 (*n =* 2)

*^a^* Measurements realised in the presence of 4 → 30 µM xanthine.

## Data Availability

Data is contained within the article or supplementary material.

## Supplementary Materials

The following supporting information can be downloaded at: https://www.mdpi.com/article/10.3390/antiox12040825/s1, Table S1: Compounds **ALS-1**→**36** identified by virtual screening.
