# Supplementary material for: Novel Reversible Inhibitors of Xanthine Oxidase Targeting the Active Site of the Enzyme
Source: Antioxidants (Basel). 2023 Mar 28;12(4):825. doi: 10.3390/antiox12040825 (PMC10135315; doi:10.3390/antiox12040825)
Supplement: Supplementary file 1 [file antioxidants-12-00825-s001.zip › antioxidants-2288625-supplementary.pdf]

Supplementary Materials

Novel reversible inhibitors of xanthine oxidase targeting the active site of the enzyme

Rosario Rullo[1,2,†], Carmen Cerchia[3,†], Rosarita Nasso[4], Virgilio Romanelli[3], Emmanuele De Vendittis[2], Mariorosario Masullo[4,*] and Antonio Lavecchia[3,*]

[1] Institute for the Animal Production Systems in the Mediterranean Environment, CNR, 80055 Portici (NA), Italy; rosario.rullo@cnr.it (R.R.)
[2] Department of Molecular Medicine and Medical Biotechnology, University of Naples Federico II, 80131 Naples, Italy; devendit@unina.it (E.D.V.)
[3] Department of Pharmacy, "Drug Discovery" Laboratory, University of Naples Federico II, 80131 Naples, Italy; carmen.cerchia@unina.it (C.C.); virgilio.romanelli@unina.it (V.R.); antonio.lavecchia@unina.it (A.L.)
[4] Department of Human Movement Sciences and Wellness, University of Naples "Parthenope", 80133 Naples, Italy; rosaritanasso@gmail.com (R.N.); mario.masullo@uniparthenope.it (M.M.)

* Correspondence: antonio.lavecchia@unina.it; Phone: +39-081-678613/623 (A.L.); mario.masullo@uniparthenope.it; Phone +39-081-5474676 (M.M).

† R.R. and C.C. equally contributed to this paper.

Table S1. Compounds ALS-1→36 identified by virtual screening [46].

N.	Approach	ID	Structure
**1**	LBVS	ALS-1	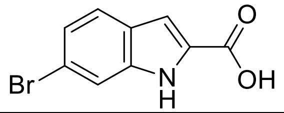
**2**	LBVS	ALS-2	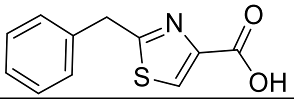
**3**	LBVS	ALS-3	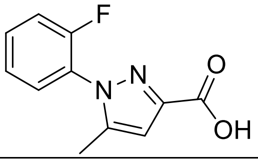
**4**	LBVS	ALS-4	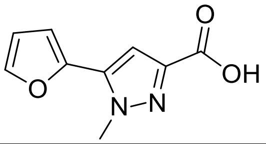
**5**	LBVS	ALS-5	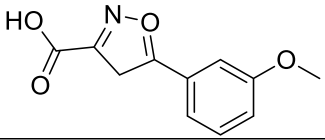
**6**	LBVS	ALS-6	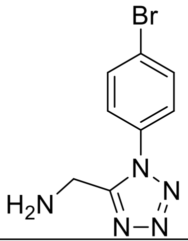
**7**	LBVS	ALS-7	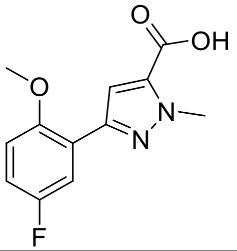
**8**	LBVS	ALS-8	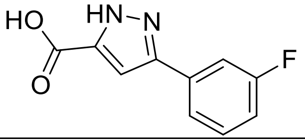
**9**	LBVS	ALS-9	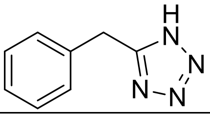
**10**	LBVS	ALS-10	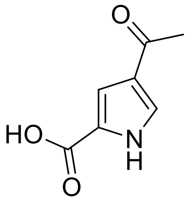

**11**	LBVS	ALS-11	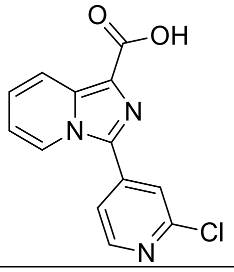
**12**	LBVS	ALS-12	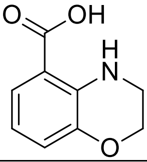
**13**	LBVS	ALS-13	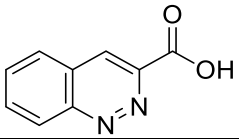
**14**	LBVS	ALS-14	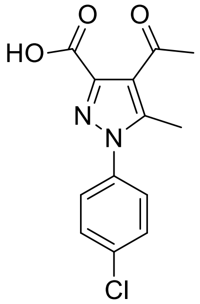
**15**	LBVS	ALS-15	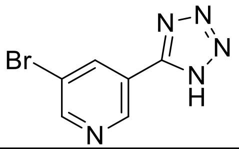
**16**	LBVS	ALS-16	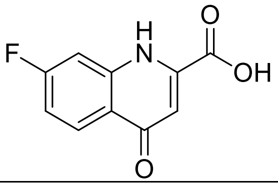
**17**	LBVS	ALS-17	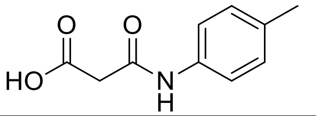
**18**	SBVS	ALS-18	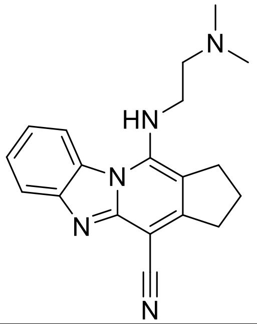
**19**	LBVS	ALS-19	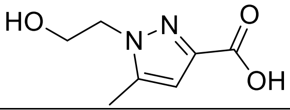

**20**	LBVS	ALS-20	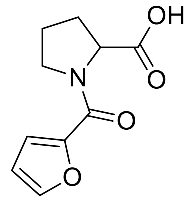
**21**	SBVS	ALS-21	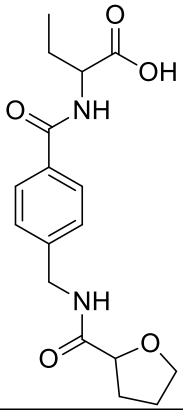
**22**	LBVS	ALS-22	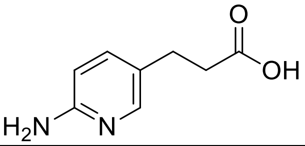
**23**	LBVS	ALS-23	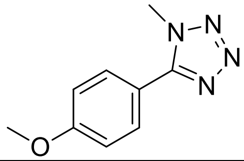
**24**	LBVS	ALS-24	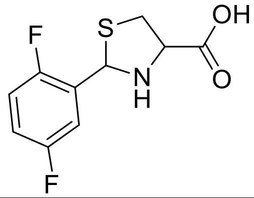
**25**	LBVS	ALS-25	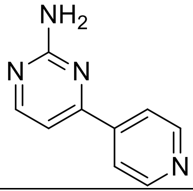
**26**	LBVS	ALS-26	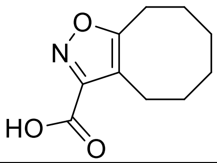
**27**	LBVS	ALS-27	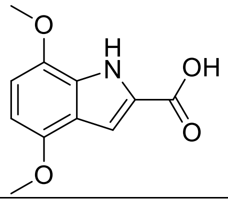
**28**	LBVS	ALS-28	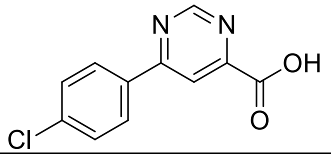

**29**	SBVS	ALS-29	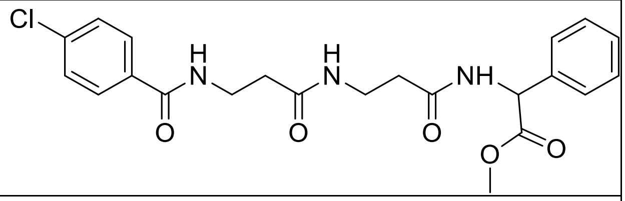
**30**	SBVS	ALS-30	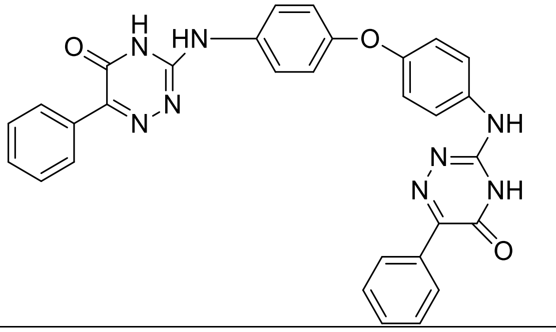
**31**	SBVS	ALS-31	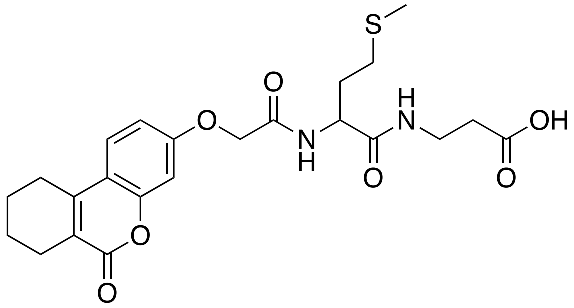
**32**	SBVS	ALS-32	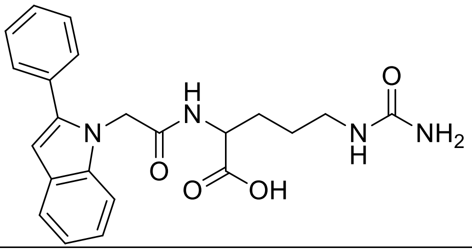
**33**	SBVS	ALS-33	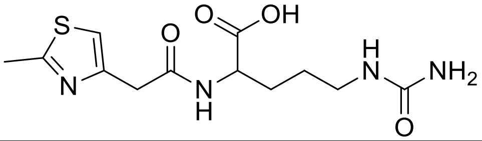
**34**	SBVS	ALS-34	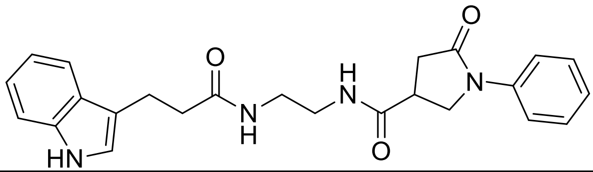
**35**	SBVS	ALS-35	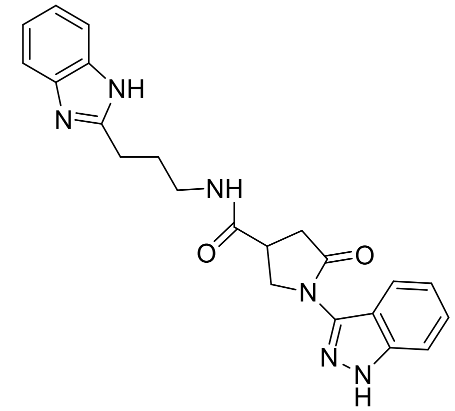

**36**	SBVS	ALS-36	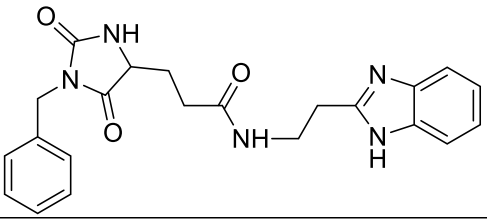
